# Conservative surgical treatment with fertility preservation in a young adult with *NTRK* rearranged spindle cell neoplasm of the uterine cervix

**DOI:** 10.1016/j.gore.2023.101233

**Published:** 2023-06-24

**Authors:** Marco M. Bühler, Hanna Honcharova-Biletska, Chantal Pauli, Dimitrios Chronas, Kristina Bolten

**Affiliations:** a Department of Pathology and Molecular Pathology, University Hospital of Zurich, Zurich, Switzerland; b Medical Faculty, University of Zurich, Zurich, Switzerland; c Gynecology and Gynecological Oncology, Department of Women’s Health, Zollikerberg Hospital, Zollikerberg, Switzerland

**Keywords:** *NTRK* fusion, Cervical sarcoma, *NTRK* rearranged tumor, Conservative surgery

## Abstract

In depth molecular studies are constantly expanding our understanding and refining the classification of gynecological neoplasms. *NTRK* rearranged spindle cell neoplasms of the lower genital tract are an emerging entity, of particular interest due to possible targeted treatment with selective kinase inhibitors. Nonetheless, surgery remains the initial treatment of choice. We present the case of a 24-year-old patient suffering from a *NTRK* rearranged spindle cell neoplasm of the uterine cervix which was treated with a fertility preserving conservative surgical approach.

## Highlights

• *NTRK* rearranged spindle cell neoplasms of the lower female genital tract are an emerging entity.
• Characteristic histomorphological changes can give hints for the correct diagnosis.
• We present a case where a fertility preserving local surgical treatment was performed.

## 1 Introduction

Mesenchymal neoplasms of the lower genital tract account for multiple entities and usually originate from the subepithelial stromal cells or the mesenchyme. As these tumors can share some morphologic overlap and similar immunohistochemical features, the diagnosis relies on clinical, morphological and radiological correlations. Previously mesenchymal tumors were mainly classified according to their putative cellular origin (e.g. smooth muscle, adipose tissue, nerve sheath), however recent advances in molecular analysis have uncovered several new entities which are characterized by genetic alterations such as kinase-related fusions. One of these emerging entities are *NTRK* rearranged spindle cell tumors or *NTRK* rearranged sarcomas, which show characteristic morphological and immunophenotypical patterns and present novel therapeutic possibilities.

## 2 Case description

A 24-year-old patient presented at our hospital for a resection of a cervical polyp that had been diagnosed during her annual gynaecological check-up a day earlier. She reported a slight increase in vaginal discharge as her sole symptom. Vaginal examination showed a polyp visible in the distended external *os* of the cervix which bled on slight touch. Intraoperatively, the polyp appeared solid and bilobated, again bleeding on the slightest touch. Its stem was very robust and the polyp could not be removed by twisting and was therefore resected as close as possible to its insertion in the cervical canal. The hysteroscopy showed a normal uterine cavum and cervical canal, although the very distal part and site of attachment was not assessable with the hysteroscope. An endometrial biopsy was performed as well as a cervical curettage.

**Fig. 1:**
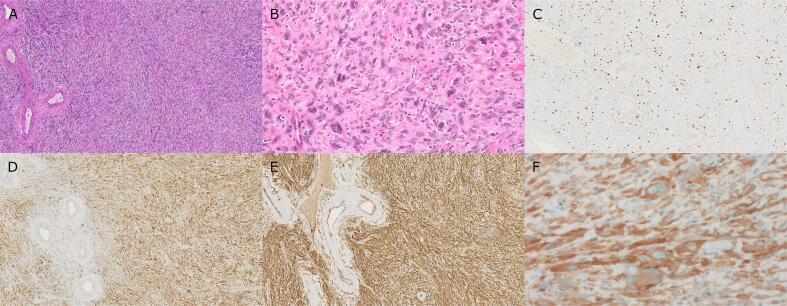
**Histopathological findings of cervical polyp.**A Low power magnification shows a tumor composed of spindle shaped cells with ovaloid nuclei. Marked perivascular and light stromal hyalinosis (H&E, 100x).B High power magnification shows focal atypia of tumor cells with enlarged nuclei (H&E, 400x). C Ki67 immunohistochemistry shows a Ki67 Proliferation index of around 15% (100x).D S100 immunohistochemistry shows diffuse positivity in tumor cells (100x). E CD34 immunohistochemistry shows diffuse positivity in tumor cells (100x). F panTRK immunohistochemistry (clone EPR17341) shows a cytoplasmic positivity in tumor cells (400x).

Macroscopically the resection specimen had a diameter of 2.2 cm and presented a tan cut surface. Histological analysis showed an ill-delimitated cellular proliferation below the endocervical surface. The tumor was composed of spindle shaped cells with ovoid nuclei with a variable degree of atypia (Fig. 1A-B). Notably, focal perivascular hyalinization was observed (Fig. 1A), as well as a discrete accompanying lymphocytic infiltrates. Immunohistochemical analysis showed diffuse positive CD34 and S100 expression (Fig. 1D-E), while there was no expression of cytokeratins (AE1/AE3), melan-A, SMA, desmin, caldesmon, calponin, CD117, DOG1, STAT6, ALK, ROS1, CD99 and ERG. The proliferation index Ki-67 amounted up to 15% (Fig. 1C), mainly highlighting the more atypical appearing cells. Pan-Trk immunohistochemistry (clone EPR17341) showed a clear cytoplasmatic positivity without nuclear positivity (Fig. 1F). RNA sequencing (Thermo Fisher Oncomine Comprehensive Assay) revealed a *TPM3::NTRK1* fusion (*TPM3* exon 7 and *NTRK1* exon 10). A diagnosis of *NTRK* rearranged spindle cell tumor was rendered, confirmed by a reference soft tissue pathologist. Atypical spindle cells were present at the resection margin and in the endocervical curettage material, accounting for a R1 resection status.

A PET-CT showed no signs of abnormal metabolic activity. Due to the patient’s young age and her wish to preserve fertility, she rejected the hysterectomy recommended by the tumor board and underwent a cervix conisation and hysteroscopy as well as abrasion instead. This procedure was performed two months after polyp resection. Neither the cone nor the endometrial biopsy showed any residues of the neoplastic cells, however the endocervical curettage displayed one small fragment with immunohistochemical positivity for CD34, S100 and pan-TRK in the subepithelial layer, thus technically implying a R1-resection. Hysterectomy was again discussed but rejected by the patient and a stringent clinical follow up with transvaginal ultrasound every 3 months as well as 6-monthly MRIs of the pelvis followed. After more than 36 months, there have been no signs of recurrence. Psychologic counselling accompanied the patient throughout this time, especially in the early phase after diagnosis. She also desired to explore options of complementary medicine and had injections of European mistletoe extract for the first 30 months.

## 3 Discussion

The reported case highlights the difficulties encountered when treating patients with newly defined emerging entities, where scientific literature with information on clinical course is limited. Although a majority of cases of *NTRK* rearranged spindle cell tumors seem to have a limited aggressive clinical course, few cases with local and systemic recurrence have been reported (Croce et al., 2019, Suurmeijer et al., 2018). From an epidemiological standpoint this neoplasm seems to mainly arise in young patients (Chiang et al., 2018, Croce et al., 2019, Mills et al., 2011), which poses the problem of infertility caused by a radical surgical treatment (i.e. hysterectomy). A recent review (Costigan et al., 2022) of the largest series to date (15 cases, including the present case) identified adverse prognostic factors: high mitotic index (greater than8 mitoses/10 high power field), lymphovascular invasion, necrosis and *NTRK3* fusions, all of which were not found in the presented case. An adverse outcome was seen in 3 cases of the series; all of them showed at least one of the prognostic factors mentioned above. Our report shows an example of a conservative surgical approach with the goal of fertility preservation. Similarly, a recent report described a case with the same *TPM3::NTRK1* fusion in a 13-year-old patient, where neoadjuvant TRK-inhibitor was used to down-size the tumor in volume, allowing a subsequent local excision (Goulding et al., 2021). If proven effective in more cases, this could represent a promising treatment strategy to reduce the need for radical surgical excision.

Thanks to the advent and availability of high throughput sequencing, the landscape of mesenchymal tumors with recurrent genetic abnormalities is expanding. In addition, pan-Trk immunohistochemistry has been shown to be a sensitive biomarker, which can be used for screening or when molecular testing for *NTRK* rearrangements is unavailable (Haberecker et al., 2023). In the past tumors with *NTRK* rearrangement and S100 positivity in this location have also been described as malignant peripheral nerve sheath tumors (Chuang et al., 2020, Wells et al., 2019), but diffuse S100 positivity and lack of SOX10 expression argues against this diagnosis. These tumors should be classified as *NTRK* rearranged spindle cell neoplasms according to the current WHO classification of tumors of the female genital tract nomenclature (5th edition) (McCluggage et al., 2022), although in the more recent literature the term *NTRK*-rearranged uterine sarcoma is favored, due to documented cases with malignant behavior. In case of tumor recurrence, systemic treatment with *NTRK* inhibitors has been shown to be highly effective in a wide variety of tumor entities (Doebele et al., 2020, Drilon et al., 2018), providing an option for targeted treatment.

## 4 Conclusion

*NTRK* rearranged spindle cell tumors of the lower genital tract (or *NTRK* rearranged uterine sarcomas) are an emerging entity recently recognized in the WHO tumor classification. These tumors are preferentially located in the cervix and are mostly limited to the uterus at time of diagnosis (Croce et al., 2020). Because of documented cases of recurrence and patients who died of the disease, they should be regarded as low-grade sarcomas and the clinical management should be discussed in interdisciplinary tumor boards. It is important to correctly classify these neoplasms, as effective *NTRK* targeting treatments are available. The key to the correct diagnosis lies in careful histopathologic examination, including immunohistochemical and molecular analysis. There is a need for further studies to elucidate the clinical course of this entity to inform adequate treatment possibilities. This is of special importance because these tumors often arise in young women (mean age 31 years), where a radical surgical treatment leads to infertility.

Written informed consent was obtained from the patient prior to publication of this case report.

## CRediT authorship contribution statement

**Marco M. Bühler:** Conceptualization, Writing – original draft. **Hanna Honcharova-Biletska:** Formal analysis, Writing – review & editing. **Chantal Pauli:** Writing – review & editing. **Dimitrios Chronas:** Writing – review & editing. **Kristina Bolten:** Conceptualization, Writing – original draft.

## Declaration of Competing Interest

The authors declare that they have no known competing financial interests or personal relationships that could have appeared to influence the work reported in this paper.
